# Thrombotic Thrombocytopenic Purpura: A Case Presenting with Acute Ischemic Colitis

**DOI:** 10.1155/2013/592930

**Published:** 2013-05-07

**Authors:** Joseph R. H. See, Tarek Sabagh, Christopher J. Barde

**Affiliations:** Wright State University Boonshoft School of Medicine, MVHG Suite 6250 30 E. Apple Street, Dayton, OH 45409, USA

## Abstract

Thrombotic thrombocytopenic purpura (TTP) consists of the pentad of thrombocytopenia, hemolytic anemia, fever, neurologic abnormalities, and renal disease. We present a case report of acute TTP following a bout of ischemic colitis. This report reminds the clinician that ischemic colitis can be an atypical presentation of TTP. The prompt recognition and treatment of this disease process resulted in a good prognosis for our patient.

## 1. Introduction

Thrombotic thrombocytopenic purpura (TTP) was first described by Moschowitz in 1925 as a disease characterized by the pathological findings of hyaline thrombi in many organs [1]. In its classic form, it consists of the pentad of thrombocytopenia, microangiopathic hemolytic anemia, neurologic abnormalities, fever, and renal disease. Currently unexplained thrombocytopenia and microangiopathic hemolytic anemia are the two criteria required to establish the diagnosis of thrombotic microangiopathy and initiate treatment [2, 3]. Ischemic colitis as a manifestation of Moschokowitz's syndrome was first reported in 1989 and other atypical manifestations of TTP have ranged from acute respiratory distress syndrome, pancreatitis, hepatitis, peripheral digital ischemia to non-occlusive mesenteric ischemia [4–7]. This case report describes the occurrence of an atypical presentation of thrombotic thrombocytopenic purpura presenting with ischemic colitis.

## 2. Case Presentation

A 70-year-old white female with a 2-week history of vertigo, nausea, and bilious vomiting presented to the emergency room after having 2 days of bloody diarrhea and diffuse abdominal pain. She had a total of 12 episodes of diarrhea. The patient complained of fevers, chills, fatigue, poor oral intake, and weight loss. There were no precipitating or relieving factors. She denied any recent travel, sick contacts, pets, raw foods, seafood exposure, antibiotic use, coffee ground emesis, hematemesis, or tenesmus. Her past medical history included hyperlipidemia, lactose intolerance, and irritable bowel syndrome with a normal colonoscopy three years ago. There was no history of colon cancer. 

**Figure 1:**
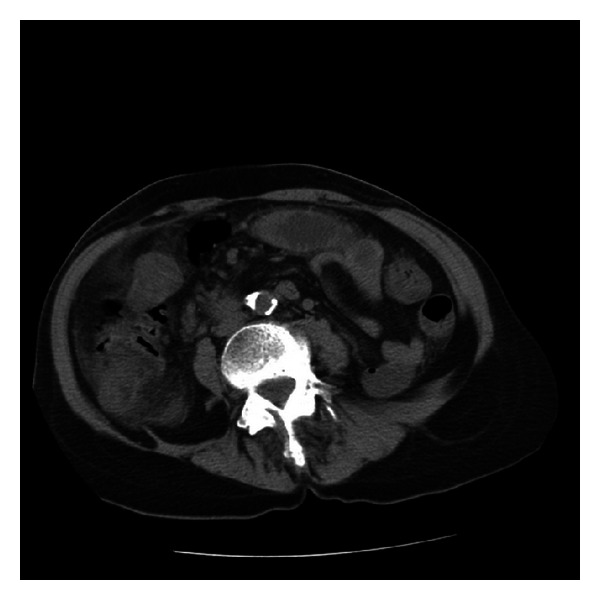
Acute colitis in the ascending and transverse colon.

Upon admission, the patient was afebrile 37.1C; pulse rate 117 beats per minute; respirations 26 breaths per minute; and blood pressure 171/96. She appeared to be in some discomfort. Her cardiac exam revealed sinus tachycardia without a murmur. There was mild abdominal distention with diffuse tenderness and decreased bowel sounds. There was no peripheral edema. Laboratory examination revealed a white blood cell count of 18,700/*μ*L with a differential of 83% segmented neutrophils; hemoglobin of 15.9 g/dL with a mean cell volume of 91.3 fL, platelet count of 206,000/*μ*L. Blood chemistry evaluation revealed a normal creatinine, normal liver function tests except for an indirect bilirubin of 1.5 mg/dL and AST of 75 U/L. Her LDH was elevated at 961 IU/L. Lactic acid level and lipase were all within normal limits. Her urine was concentrated and revealed ketones. CT scan demonstrated acute colitis in the ascending and partial transverse colon (Figure 1). The patient was admitted and started on ciprofloxacin and metronidazole. Blood and stool cultures were obtained. She was placed on omeprazole for gastrointestinal prophylaxis.

**Figure 2:**
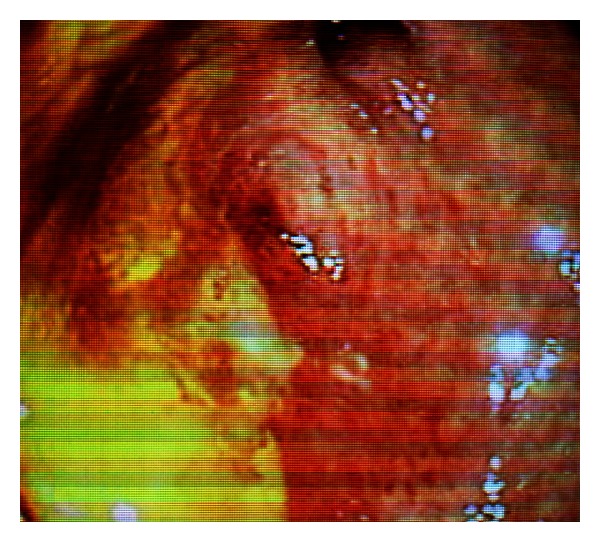
Mucosal edema with friability and ulceration on colonoscopy.

The patient underwent a colonoscopy on hospital day 2 which revealed multiple diverticuli, evidence of mucosal edema with friability and ulceration from the transverse colon to sigmoid colon (Figure 2). The colonoscopy was not advanced beyond the transverse colon because of patient discomfort. Random colon biopsies were obtained. Her metronidazole was changed from intravenous to parental and clear liquids were started in the morning.

**Figure 3:**
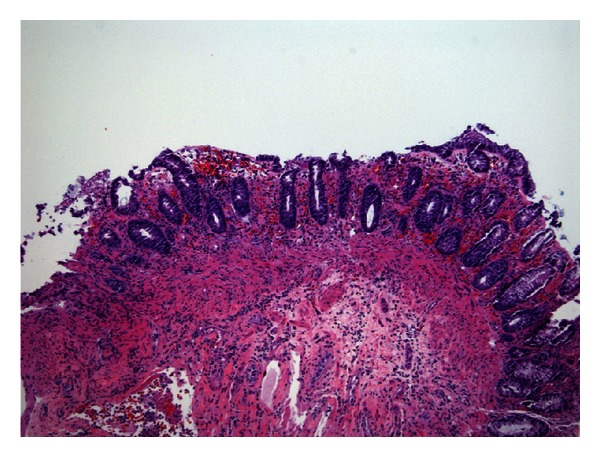
Colonic biopsy at medium magnification (100x, H&E stain) showing features characteristic of ischemic injury, including crypt atrophy, loss of cytoplasmic mucin, nuclear hyperchromasia, and absence or shedding of the luminal surface epithelium.

Her colonic biopsies came back and were consistent with ischemic colitis (Figure 3). A CT angiography of the abdomen and pelvis showed no evidence of mesenteric arterial insufficiency. There was also improvement in the wall thickening previously seen in the right and transverse colon compared to the CT on admission. *Saccharomyces cerevisiae* antibodies (ASCA), antinuclear antibody (ANA), myeloperoxidase (MPO), and stools studies were negative. Her stools studies also came back negative for *E. Coli 0157:H7, clostridium difficile, salmonella, Yersinia, and Campylobacter*. The patient continued to improve and her bloody diarrhea resolved.

**Figure 4:**
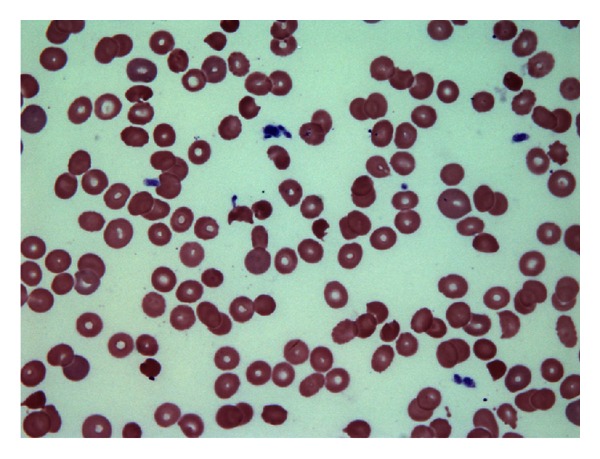
Peripheral blood (Wright Stain, 1000x) showing schistocytes, characteristic of microangiopathic hemolytic anemia.

On hospital day 3 the platelet count was 76,000/*μ*L with hemoglobin of 12.4 g/dL. On hospital day 7 the platelet count was 39,000/*μ*L and the hemoglobin of 7.8 g/dL. Her neurological examination was unremarkable. Hematology consultation was obtained because of the thrombocytopenia. Laboratory examination revealed a decreased haptoglobin, elevated D dimer, elevated LDH, normal PT/PTT, elevated thrombin time, and increased CRP and reticulocyte count. Schistocytes were observed on the peripheral smear and bone marrow biopsy. (Figure 4) Her omeprazole was discontinued, steroids were started, and the patient underwent therapeutic plasmapheresis with plasma exchange. The patient's ADAMTS 13 came back normal. Her platelets and hemoglobin dropped towards the end of her plasmapheresis. The patient was started on rituximab. She received a total of 16 plasma exchanges, 4 doses of rituximab, and was sent home on hospital day 30.

## 3. Discussion

Our patient initially presented with bloody diarrhea and was found to have ischemic colitis which was the initial presentation of TTP. Typical childhood HUS presents with an episode of abdominal pain and bloody diarrhea that is most often due to Shiga toxin-producing enterohemorrhagic *Escherichia coli* while TTP usually affects adults. It is an acute syndrome associated with thrombi composed of platelets in multiple organ systems. 

Non-occlusive mesenteric ischemia (NOMI) as documented by the CT angiogram was the cause of these symptoms. NOMI has been reported elsewhere as a manifestation of TTP [6]. Decreased blood flow in the mesentery, increased metabolic demand and platelet thrombi occluding the capillaries of the colon likely contributed to the development of ischemic colitis. This condition tends to occur in patients with significant atherosclerotic disease. Other inciting events include aortic insufficiency, sepsis, arrhythmias, and drugs such as alpha-adrenergic agonists and cocaine use. NOMI accounts for 20–30 percent of patients with acute mesenteric ischemia [7]. The clinical manifestations vary depending on the extent and duration of ischemia [8]. Symptoms include mild abdominal pain and tenderness over the affected bowel followed by rectal bleeding or bloody diarrhea. Laboratory studies are nonspecific. Our patient only had hyperlipidemia as a risk factor for atherosclerotic disease. She did not have any history of cardiovascular disease. She presented with leukocytosis along with a normal lactic acid level.

Because of the patient's thrombocytopenia, other processes needed to be considered. This patient was also on a proton pump inhibitor (PPI) for the first seven days of her hospital stay. Drug induced thrombocytopenia is often poorly understood. In a number of cases, thrombocytopenia may be the only hematologic manifestation of drug toxicity. Aplastic anemia and TTP due to some drug-induced disorder typically result in thrombocytopenia along with other cytopenias and organ involvement. A number of drugs have been associated with TTP-HUS such as quinine, gemcitabine, oxaliplatin, pentostatin. PPI use should not be overlooked in the differential diagnosis. Omeprazole was discontinued because several case reports have revealed a possible relationship between proton-pump inhibitors, thrombocytopenia, and TTP [9–11].

Little is known about the long-term consequences of TTP. Some patients continue to have neurocognitive deficits and renal dysfunction. Our patient is potentially at risk for relapse for the next ten years [12]. Chronic complications include colonic inflammation, chronic ischemic colitis, strictures, and potential necrosis leading to perforation and potential gangrene [13–15].

Since patients rarely present with the pentad of HUS/TTP, recognition of atypical cases of TTP and prompt initiation of the management of TTP is an important factor in improved patient survival. Prior to plasma exchange, the mortality rate approaches ninety percent whereas with treatment mortality reaches twenty percent. Plasma exchange therapy depletes the circulating level of autoantibody to ADAMTS13 and very high molecular weight von Willebrand factor (VWF) multimers along with replacement of the patients missing protease. Based on her clinical response to plasmapheresis and rituximab, our patient's ischemic colitis likely represented TTP. TTP is a medical emergency that is almost always fatal if exchange plasmapheresis is not initiated early. Patients with a severe course of TTP who do fully respond to plasma exchange may benefit from rituximab. This case report reminds clinicians that ischemic colitis maybe an atypical presentation of TTP. The prompt diagnosis and treatment of this condition resulted in a good prognosis for our patient.
